# Overexpression of the leucine‐rich receptor‐like kinase gene *LRK2* increases drought tolerance and tiller number in rice

**DOI:** 10.1111/pbi.12707

**Published:** 2017-03-23

**Authors:** Junfang Kang, Jianmin Li, Shuang Gao, Chao Tian, Xiaojun Zha

**Affiliations:** ^1^ College of Chemistry and Life Sciences Zhejiang Normal University Jinhua China

**Keywords:** rice, *LRK2*, drought stress, tiller

## Abstract

Drought represents a key limiting factor of global crop distribution. Receptor‐like kinases play major roles in plant development and defence responses against stresses such as drought. In this study, *LRK2*, which encodes a leucine‐rich receptor‐like kinase, was cloned and characterized and found to be localized on the plasma membrane in rice. Promoter–GUS analysis revealed strong expression in tiller buds, roots, nodes and anthers. Transgenic plants overexpressing *LRK2* exhibited enhanced tolerance to drought stress due to an increased number of lateral roots compared with the wild type at the vegetative stage. Moreover, ectopic expression of *LRK2* seedlings resulted in increased tiller development. Yeast two‐hybrid screening and bimolecular fluorescence complementation (BiFC) indicated a possible interaction between LRK2 and elongation factor 1 alpha (OsEF1A) *in vitro*. These results suggest that *LRK2* functions as a positive regulator of the drought stress response and tiller development via increased branch development in rice. These findings will aid our understanding of branch regulation in other grasses and support improvements in rice genetics.

## Introduction

Rice (*Oryza sativa*) is one of the most important crops, feeding more than one‐third of the world's population. This major staple requires large amounts of water during growth and is therefore susceptible to drought stress. At each stage of growth, drought hinders crop development and decreases yield. Molecular genetic tools aimed at improving rice drought tolerance and maintaining production, while expanding development into regions with limited water resources is therefore important (Fernie *et al*., 2006; Pennisi, 2008).

Leucine‐rich repeat receptor‐like kinase (LRK), which belongs to the largest subfamily of kinases in plants, contains a leucine‐rich extracellular domain, a transmembrane domain and a C‐terminal intracellular kinase domain (Shiu *et al*., 2004; Walker, 1993). At least 223 *LRKs* are known in *Arabidopsis* and more than 300 in rice (Shiu *et al*., 2001). *LRKs* function in a number of developmental processes and defence responses such as meristem maintenance (Clark *et al*., 1997), cellular proliferation (Matsubayashi *et al*., 2002), brassinosteroid signalling (Li and Chory, 1997), floral organ abscission (Jinn *et al*., 2000; Taylor *et al*., 2016) and defence bacterial flagellin (Zipfel *et al*., 2006). However, known functions in rice remain limited to, for example, *ERECTA* (Shen *et al*., 2015), *Xa21* (Jiang *et al*., 2013; Song *et al*., 1997)*, OsSIK1* (Ouyang *et al*., 2010) and *FON1* (Feng *et al*., 2014), with the majority of genes yet to be elucidated.

Eukaryotic elongation factor (eEF) proteins can be divided into eEF1 and eEF2. eEF1 proteins, which can further be divided into eEF1A, eEF1Bα, eEF1Bβ and eEF1Bγ subunits, are highly conserved in a number of species (Browning, 1996). eEF1‐mediated ammonia acyl‐tRNA has also been shown to bind to ribosomes (Riis *et al*., 1990). Moreover, recent studies have shown that *eEF1A* is not only important for translation, but is also an important multifunctional protein (Ejiri, 2002; Sasikumar *et al*., 2012), playing a role in processes such as cell proliferation (Pecorari *et al*., 2009; Sanders *et al*., 1992), cell apoptosis (Byun *et al*., 2009; Shepherd *et al*., 1989; Zhang *et al*., 2015), cell morphogenesis (Gross and Kinzy, 2005) and signal transduction (Numata *et al*., 2000). However, most of these studies have focused on animals and humans, with few findings in plants. In *Arabidopsis*,* eEF1B* is associated with cell wall biosynthesis and plant development (Hossain *et al*., 2012). Moreover, the *AtEF2* gene has been shown to be involved in low temperature signalling (Guo *et al*., 2002). Since 1998, four *EF1A* genes have also been cloned in rice, but their functions have yet to be reported (Kidou and Ejiri, 1998).

Previously, an eight leucine‐rich LRK gene (*LRK1‐LRK8*) cluster, which has been shown to increase grain yield, was cloned from the rice quantitative trait locus (QTL) *qGY2‐1* (Li *et al*., 2002a). Haplotype divergence of the *LRK* locus was subsequently found to be associated with the origin and differentiation of cultivated rice. Moreover, *LRK2* was found to be highly expressed in *Oryza sativa* L. ssp. *indica var*. 9311, but was undetectable by RT‐PCR in *Oryza sativa* L. ssp. *japonica cv*. Nipponbare (He *et al*., 2006). In the present study, we cloned and characterized *LRK2* and revealed an increase in branch number and drought tolerance in *LRK2*‐overexpressing transgenic lines. *pLRK2::GUS* was largely expressed in tiller buds, nodes, roots and anthers. Furthermore, yeast two‐hybrid screening and bimolecular fluorescence complementation (BiFC) analysis suggested an interaction between *LRK2* and *OsEF1A*. The molecular mechanisms underlying the response of rice to drought was also discussed with the aim of improving crop growth under potentially adverse conditions.

## Results

### *LRK2* encodes a leucine‐rich receptor‐like kinase

**Figure 1:**
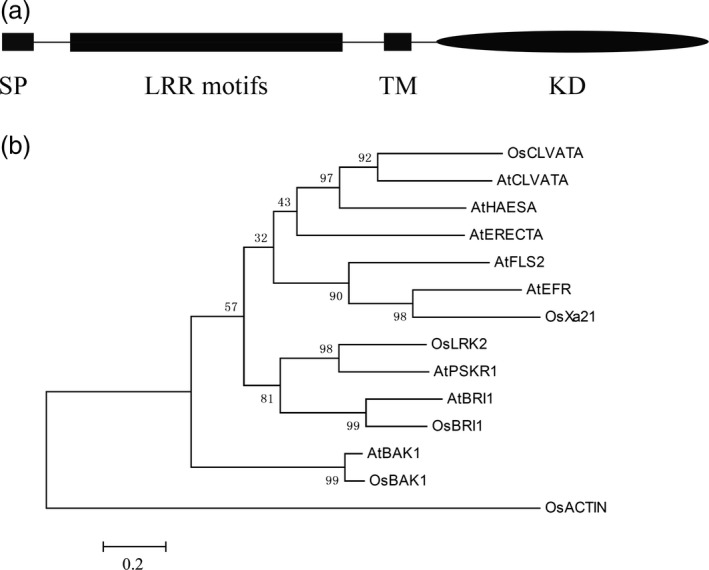
LRK2 encodes a leucine‐rich receptor‐like kinase. (a) Schematic diagram of the LRK2 protein. SP, signal peptide; LRR motif, leucine‐rich repeat region; TM, transmembrane domain; KD, intercellular kinase domain. (b) Phylogenetic analysis of deduced amino acid sequences of LRK2 compared with other homologous sequences.

LRK2 contains extracellular LRR motifs, a transmembrane domain (TM) and a cytoplasmic kinase domain (Figure 1a). To investigate the relationships between LRK2 (GenBank accession no. AY756174.4 GI:54306232) and LRK members from other plant species, phylogenetic analysis of LRKs from rice and *Arabidopsis thaliana* was performed using Clustalx1.83 and MEGA 6. Analysis involved 1000 bootstrap replicates with the following sequences: OsCLVATA: EAY84170; AtCLVATA: AAB58929; AtHAESA: XP_002869498; AtERECTA: XP_002880777; AtFLS2: AAO41929.1; AtEFR: AAL77697.1; OsX21: NC_008395.2; LRK2: AY756174.4; AtBRI1: XP_002866847.1; OsBRI1: AAK52544.1; AtBAK1: NP_567920.1; OsBAK1: EEC82980.1; and AtPSKR1:At2g02220. Based on a comparison of homologous amino acid sequences, LRK2 was found to share a close genetic relationship with phytosulfokine receptor 1 (PSKR1) from *A. thaliana* (Hartmann *et al*., 2014; Matsubayashi *et al*., 2002, 2006; Figure 1b). In addition, the function of LRK2 as a novel leucine‐rich repeat receptor‐like kinase (LRR‐RLK) was reported for the first time.

### Features of *LRK2* in rice

**Figure 2:**
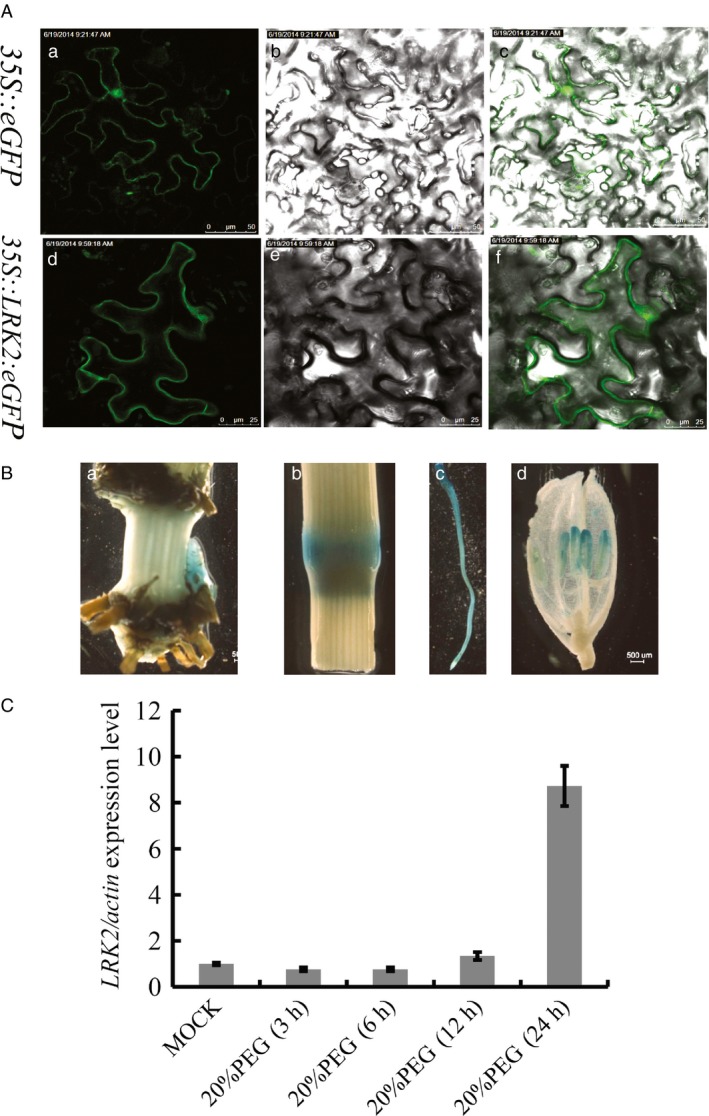
Expression patterns and subcellular localization of *LRK2* in rice. (A) Subcellular localization of *LRK2*. a,d: under the laser (430 nm); b,e: bright field; c,f: merged image; a,b,c: sections for *35S::eGFP*; d,e,f: sections for *LRK2* construction. Bars = 25 μm. (B) GUS staining of different organs in the overexpressing line: a, tiller bud; b, node; c, root; d, anther. (C) Expression analysis of *LRK2* in Nipponbare rice seedlings under drought treatment using real‐time PCR. Each column represents an average of three replicates and bars indicate the SD.

To confirm the subcellular localization of *LRK2*,* LRK2* with an enhanced *GFP* was constructed under control of the cauliflower mosaic virus 35S promoter (*35S::LRK2:eGFP*), then infiltrated into tobacco (*Nicotiana benthamiana*) leaves by *Agrobacterium*‐mediated transient transformation. The resulting construct, *35S::LRK2:eGFP*, exhibited *eGFP* expression in the plasma membrane compared with the control, *35S::eGFP*, which showed expression throughout the cell (Figure 2A). In addition, to determine expression patterns, the 2‐kb promoter region of the *LRK2* gene was cloned into the expression vector *pBIN121* with the β‐glucuronidase (*GUS*) reporter gene (Figure S1A). The construct *pLRK2:GUS* was subsequently transformed into rice (Figure S1B). A *GUS* staining assay of T2 transgenic lines revealed expression in the tiller buds, nodes, roots and anthers (Figure 2B).

To examine whether *LRK2* expression was regulated by drought stress, 7‐day‐old rice seedlings were subjected to 20% PEG6000 then harvested for RNA extraction at different time points and the transcripts of whole plants were quantified by real‐time PCR. As a result, transcript levels were found to be rapidly and strongly induced by drought (Figure 2C).

### Expression of *LRK2* in transgenic rice lines

**Figure 3:**
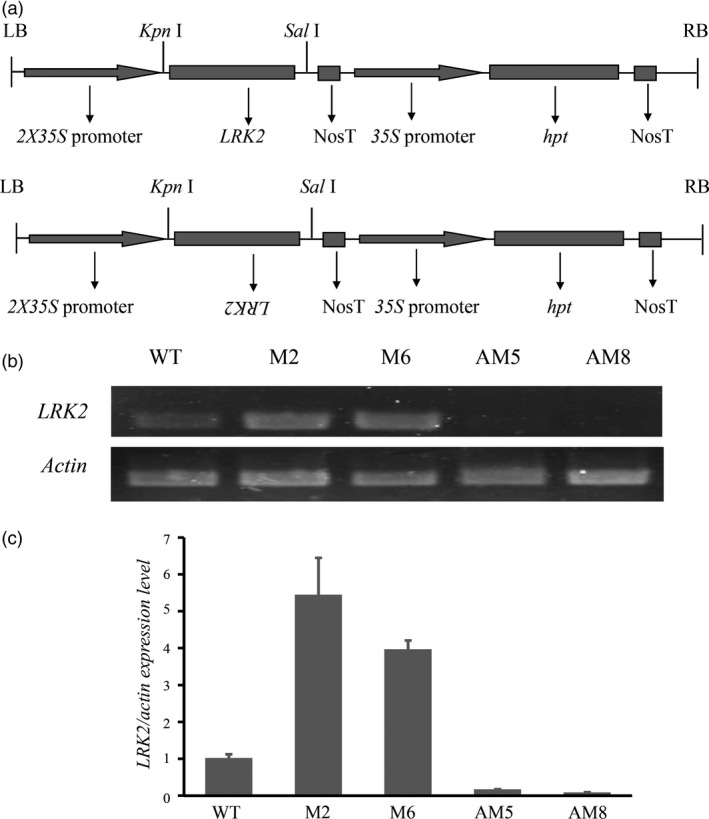
Expression of *LRK2* in transgenic rice. (a) Schematic diagram of the plant expression vectors *pCAMBIA1300‐2X35S::LRK2* and *pCAMBIA1300‐2X35S::antiLRK2* used for *LRK2* overexpression and decreased expression in transgenic plants. (b, c) Expression analysis of *LRK2* in transgenic plants using semiquantitative PCR and real‐time PCR with gene‐specific primers.

To obtain further insight into *LRK2*,* 2X35S::LRK2* and *2X35S::antiLRK2* (Figure 3a), plasmids containing the entire *LRK2* gene were introduced into Nipponbare, and transformants selected on medium containing hygromycin. The transgenic plants were simultaneously examined by PCR using genomic DNA as a template with specific primers. Eleven and eight independent transformants (T_0_) were regenerated from hygromycin‐resistant calli of the *2X35S::LRK2* and *2X35S::antiLRK2* lines, respectively (Figure S2). Four T_2_ transgenic lines plus the wild type were subsequently selected for expression analysis (M2 and M6 to represent *2X35S::LRK2*, and AM5 and AM8 for *2X35S::antiLRK2*). Expression levels during the three‐leaf stage were determined by semiquantitative and RT‐PCR. *LRK2* was strongly expressed in M2 and M6, but reduced in AM5 and AM8 (Figure 3b,c), suggesting that the *2X35S::LRK2* and *2X35S::antiLRK2* plasmids were genetically transmitted to the next generation.

### Overexpression of *LRK2* increases drought tolerance in rice

**Figure 4:**
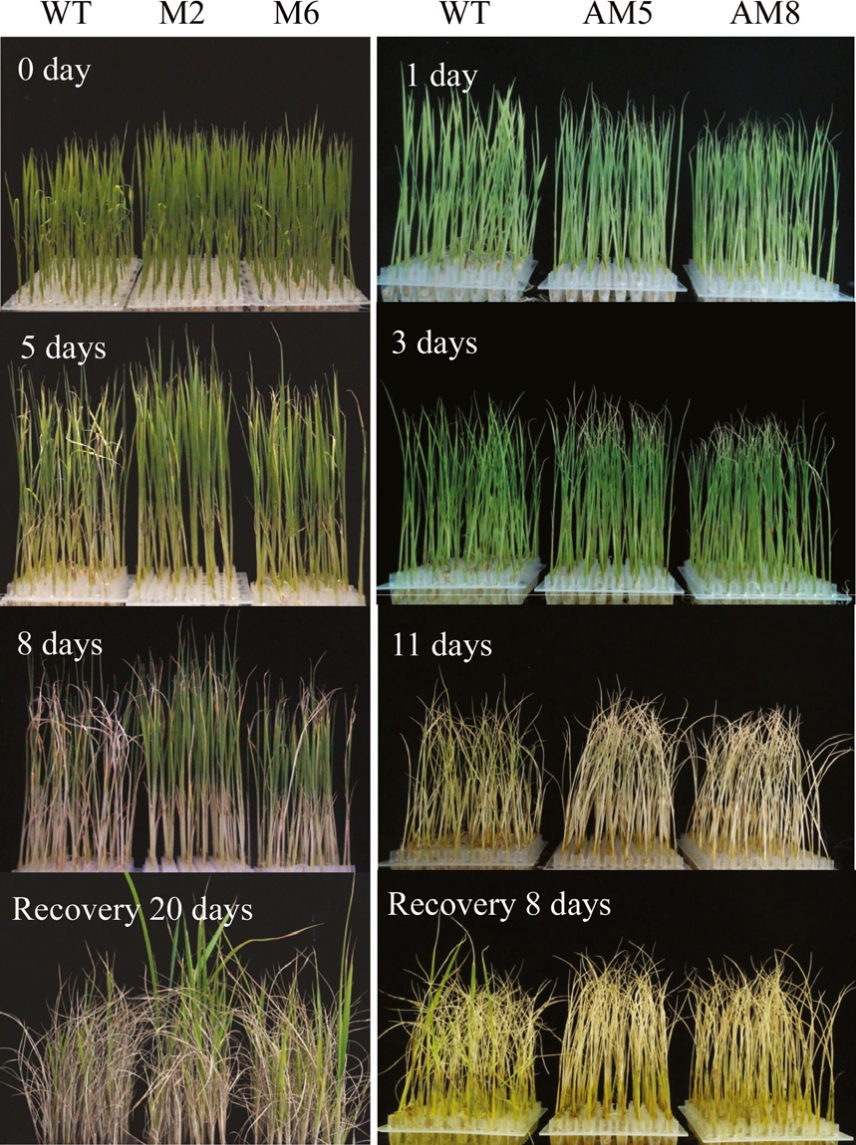
Performance of *LRK2* transgenic plants under drought stress. 0 day: Wild‐type, M2 and M6 seedlings grown for 12 days under normal conditions. 5, 8 days: Performance of wild‐type, M2 and M6 seedlings treated with 20% PEG6000 for 5 and 8 days, respectively. 1, 3, 11 days: Performance of wild‐type, AM5 and AM8 seedlings grown under drought stress for 1, 3 and 11 days, respectively. Recovery 20 days: Recovery of the treated wild‐type, M2 and M6 seedlings for 20 days. Recovery 8 days: Recovery of the treated wild‐type, AM5 and AM8 seedlings for 8 days.

LRKs reportedly regulate a number of stress responses, including drought, salinity and low temperature (Ouyang *et al*., 2010; Shen *et al*., 2015; Yang *et al*., 2014). The performance of *LRK2*‐overexpressing, *LRK2*‐antisense and wild‐type plants under drought stress conditions was therefore examined (Figure 4). Under normal conditions, all plants grew well. After 12 days, plants were cultured in 20% PEG6000 solution and then 5 days later, phenotypic changes in wild‐type and transgenic plants were observed. Leaf rolling and wilting was delayed in the M2 and M6 transgenic lines compared with the wild type, while in AM5 and AM8 symptoms of drought stress were severe. After 8‐day treatment, the M2 and M6 lines showed increased tolerance to drought stress compared with the wild type, while AM5 and AM8 remained sensitive.

**Figure 5:**
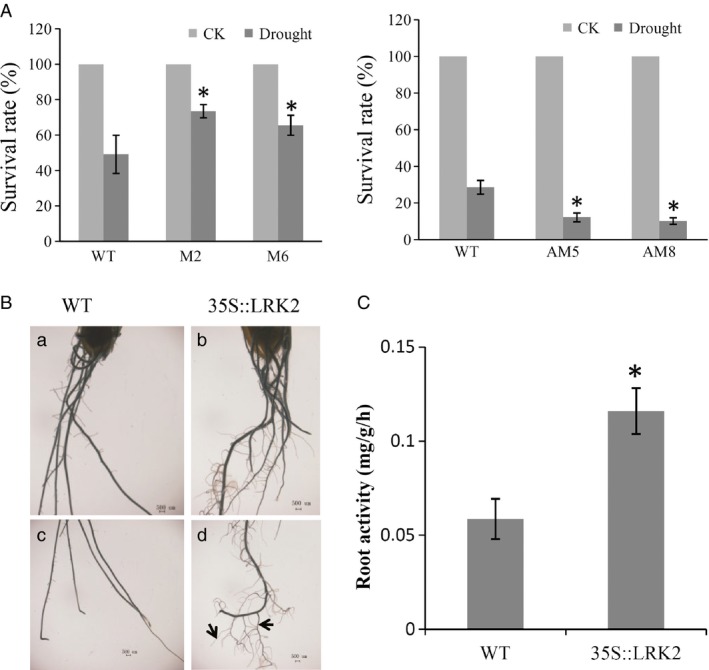
Performance of *LRK2* transgenic plants under drought stress. (A) Survival rates of the wild‐type and transgenic plants after recovery. (B) (a–d) Root characters of the plants under drought stress. Arrows indicate the large and small lateral roots. Scale bars: 500 μm. (C) Root activity of the wild‐type and *LRK2*‐overexpressing lines (LRK2). *, *P *< 0.05, *t*‐test.

Following drought treatment, the plants were watered to induce recovery, and then, the growth status was examined. M2 and M6 recovered and grew more vigorously than the wild type, with survival rates of 73.5% and 65.5%, respectively, compared with 49.2%. In contrast, only 12.2% of the AM5 and 10.2% of the AM8 plants recovered, compared with 28.6% of the wild type. All values were significantly different (*P* < 0.05, *t*‐test; Figure 5A), suggesting that *LRK2* positively regulates the drought stress response in rice.

Large root systems are known to be more conducive to extraction of water from deep soil layers compared with small root systems. The root structures of transgenic and wild‐type plants after drought treatment were therefore examined under a stereoscopic microscope. The transgenic plants had significantly more lateral roots and a larger overall root system than the wild type (Figure 5B). Root activity in rice at different stages directly affects plant growth. In this study, root activity appeared higher in *LRK2*‐overexpressing lines compared with the wild type (Figure 5C). These results suggest that increased root activity in the *LRK2*‐overexpressing lines may be one reason for the increase in lateral root development.

To analyse the expression of genes involved in drought tolerance, expression levels of 9‐cis‐epoxycarotenoid dioxygenase 1 (*NCED1*), *NCED2*, plasma membrane intrinsic protein 2;3 (*PIP2;3*) and ABA‐responsive element binding factor (*ABF*) were detected using real‐time PCR in wild‐type and transgenic seedlings under drought (20% PEG6000) for 12 h (Hatmi *et al*., 2015; Redillas *et al*., 2012; Shi *et al*., 2015; Wu *et al*., 2015; Yoshida *et al*., 2010). The data showed that *PIP2;3* was strongly expressed in *LRK2* overexpression lines (Figure S3).

### Effect of *LRK2* on rice tiller development

**Figure 6:**
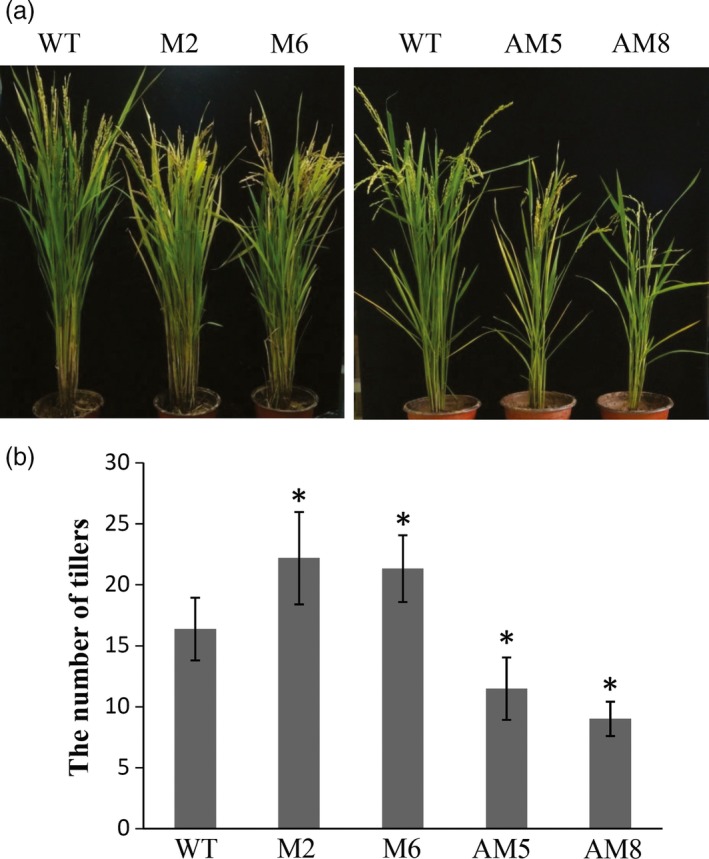
Effects of *LRK2* in transgenic plants. (a) Comparison of wild‐type and transgenic lines at the flowering stage. (b) Number of panicles per plant in the wild‐type and transgenic plants. *, *P *< 0.05, *t*‐test.

To investigate the effect of *LRK2* on yield traits, 14 transgenic lines were analysed (M1 to M6, AM1 to AM8; 20 individual plants per line). After cultivating transgenic lines and Nipponbare wild‐type plants under identical conditions, the following yield components were examined: numbers of tillers per plant, grains on the main panicle, grains per panicle and grains per plant (Table 1). The tiller is a specialized grain‐bearing branch that forms on unelongated basal internodes. At the tillering stage, the *LRK2*‐overexpressing lines exhibited increased tiller development (Figure S4A). At maturity, M2 and M6 produced 36% and 32% more panicles than the wild type, respectively, while AM5 and AM8 showed a decrease in panicles of 29.7% and 44.9% compared with the wild type, respectively (Table 1, Figures 6, S4B). The number of grains on the main panicle increased in overexpressing lines compared with the wild type (M2: 8.57%; M6: 5.46%), while the number of grains per panicle showed a slight decrease (M2: 10.13%; M6: 18.79%). In contrast, the number of grains per plant increased in the overexpressing lines (M2: 29.9%; M6; 25.3%) compared with the wild type (Table 1, Figure S4C). The number of grains per panicle is affected by the number of primary, secondary and sometimes higher‐order panicle branches. The number of primary and secondary panicle branches was therefore counted. The overexpressing lines showed a slight increase in the number of secondary branches on the main panicle compared with the wild type (M2: 7.86%; M6: 6.46%); however, the number of primary branches did not significantly differ (Table 2, Figure S4C). These results suggest that *LRK2* improves rice tiller number and the number of grains per plant.

**Table 1:** Yield components of wild‐type (WT) and transgenic plants M2, M6, AM5 and AM8

Plant line	Number of tillers per plant	Number of grains on the main panicle	Number of grains per panicle	Number of grains per plant
WT	16.38 ± 3.57	85.91 ± 4.9	76.89 ± 6.37	1036.63 ± 115.01
M2	22.28 ± 4.79	93.27 ± 5.4	69.10 ± 4.58	1347.5 ± 156.81
M6	21.34 ± 5.73	90.6 ± 5.64	62.44 ± 8.26	1298.53 ± 104.28
AM5	11.5 ± 2.56	77.56 ± 3.3	52.56 ± 8.3	752.23 ± 82.02
AM8	9.02 ± 1.41	75.13 ± 4.5	50.13 ± 7.5	612.58 ± 98.3
*P* value	*P *< 0.05	*P *< 0.05	*P *< 0.05	*P *< 0.05

Data were obtained from random samples at maturity and represent the mean ± SD of 20 individuals.

**Table 2:** Number of branches on the main panicle of wild‐type (WT) and transgenic lines M2 and M6

Plant lines	Primary branches	Secondary branches
WT	7.45 ± 0.82	15.64 ± 3.44
M2	7.93 ± 1.1	16.87 ± 2.33^a^
M6	7.54 ± 0.9	16.65 ± 2.28^a^

Data were obtained from random samples at maturity and represent the mean ±SD of 20 individuals.

a *P *< 0.05, *t*‐test.

### Interaction between LRK2 and eukaryotic elongation factor 1A

**Figure 7:**
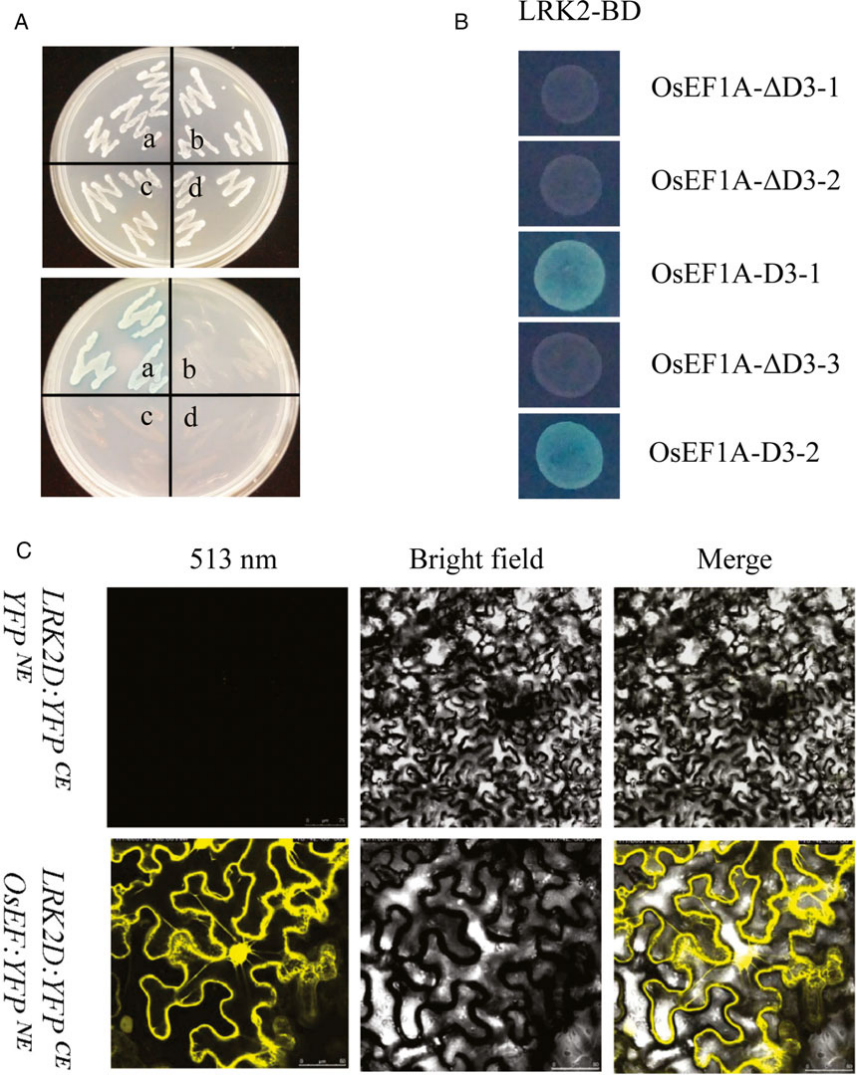
Interaction between LRK2D and OsEF1A *in vitro*. (A) Interaction between OsEF1A and the LRK2 kinase domain as bait in a yeast two‐hybrid system. Photograph shows the growth behaviour of transformants on SD/Leu‐Trp medium (upper) and SD/Leu‐Trp‐His‐/50 mM3‐AT medium (lower). The yeast cells harboured various pairs of plasmids: a. *pGBKT7‐LRK2D* with *pGADT7‐OsEF1A*; b. *pGBKT7* with *pGADT7‐OsEF1A*; c. *pGBKT7‐LRK2D* with *pGADT7*; d. *pGBKT7* with *pGADT7*. (B) The carboxyl domain of OsEF1A was found sufficient, and necessary, for interaction with the LRK2 kinase domain in yeast. Residues of OsEF1A present in the constructs were 1 to 230aa (OsEF1A‐ΔD3‐1), 231 to 320aa (OsEF1A‐ΔD3‐2), 321 to 447aa (OsEF1A‐D3‐1), 1 to 320aa (OsEF1A‐ΔD3‐3), 231 to 447aa (OsEF1A‐D3‐2). (C) Interaction between LRK2D and OsEF1A as examined by BiFC. The indicated constructs were transformed into leaves of *Nicotiana benthamiana*. Scale bars in upper images: 20 mm. Scale bars in lower images: 50 mm.

To identify interactors of LRK2, LRK2 kinase domain (*LRK2D*) was used as bait in yeast two‐hybrid analysis. As a result, six potential interacting molecules were identified. Nucleotide sequence analysis further revealed one cDNA fragment encoding the eukaryotic elongation factor *OsEF1A* (GenBank Accession no. GQ848073.1), which encodes a protein involved in protein synthesis and cell proliferation. To confirm the direct interaction between LRK2 and OsEF1A, a yeast two‐hybrid assay was performed via cotransformation of the *pGBKT7‐LRK2D* bait construct and full‐length *pGADT7‐OsEF1A*. As expected, only yeast cells harbouring both *LRK2D* and *OsEF1A* grew vigorously on both SD/Leu‐Trp‐ and SD/Leu‐Trp‐His‐/50 mm 3‐amino‐1,2,4‐triazole (3‐AT) plus X‐gal media. In contrast, the wild type grew well on SD/Leu‐Trp‐ but not SD/Leu‐Trp‐His‐/50 mm 3‐AT/ X‐gal medium (Figure 7A). *pGBKT7‐LRK2D* and a truncated version of *OsEF1A* were also cotransformed with *pGADT7*, revealing that the C‐terminal region alone is sufficient, and indeed necessary, for binding with the LRK2 kinase domain (Figure 7B). To further verify the interaction between LRK2D and OsEF1A, BiFC analysis of *Nicotiana benthamiana* leaves was performed (Figure 7C). Under 513 nm illumination, cells harbouring *LRK2D:YEP* ^*CE*^ and *OsEF1A:YEP* ^*NE*^ emitted yellow fluorescence, confirming the interaction between OsEF1A and LRK2.

## Discussion

It is predicted that global climate change will increase temperatures, alter geographical patterns of rainfall and increase the frequency of extreme climatic events (Harrison *et al*., 2014). Drought is a major constraint of crop development and production worldwide. Accordingly, a number of studies have suggested that overexpression of stress‐related genes may help improve drought tolerance in cereal crops (Cheng *et al*., 2016; Uga *et al*., 2013). In this study, a new rice leucine‐rich repeat receptor‐like kinase, *LRK2*, was found to have the ability to increase drought stress by promoting root growth and significantly increasing tiller number, while reducing plant height (Figures 6 and S4; Table S1). The data indicated that the *LRK2* gene encodes a protein localized to the plasma membrane, and expressed in tiller buds, nodes, roots and anthers (Figure 2). These results suggest that the *LRK2* gene is essential for stable and adequate crop production in drought‐prone areas.

The mature rice fibrous root system is composed of adventitious and lateral roots. Adventitious root branching results in both large and small lateral roots (Coudert *et al*., 2010), which are essential for water and nutrient uptake and critical for increased yield under stress (Atkinson *et al*., 2014; Coudert *et al*., 2010). In rice, *DRO1* was previously found to be negatively regulated by auxin and involved in cell elongation, resulting in an enlarged root system and increased drought avoidance (Uga *et al*., 2013). Moreover, rice *OsAHP1* and *OsAHP2* knockdown plants were previously found to exhibit phenotypes representative of a deficiency in cytokinin signalling, including enhanced lateral root growth and resistant to osmotic stress compared with wild‐type plants (Sun *et al*., 2014). Moreover, ectopic *OCI* expression was found to increase the lateral root density and drought tolerance in *Arabidopsis* and soya bean (Quain *et al*., 2014). In the present study, *LRK2* overexpression resulted in an improved root system with an increased number of both large and small lateral roots compared with the wild type. This is one reason for the increased drought tolerance in *LRK2*‐overexpressing lines. The 2,3,5‐triphenyltetrazolium chloride (TTC) test was used here to determine root activity in the *LRK2*‐overexpressing plants (Hu *et al*., 2016; Steponkus and Lanphear, 1967). Roots of the transgenic plants were significantly more active than those of the wild‐type plants (Figure 5C), further contributing to increased drought resistance in the *LRK2*‐expressing lines. PIP2;3, one of the aquaporins, plays a crucial role in response to drought stress (Yu *et al*., 2006). SIRK1, a member of the LRK family in *Arabidopsis*, was shown to interact with and activate PIP2;3 by phosphorylation (Wu *et al*., 2013). Experiments should be undertaken to evaluate whether LRK2 can specifically phosphorylate PIP2;3.

Plant architecture, such as the structure of the roots, shoots and inflorescences, is affected by branching. Shoot branches in rice are referred to as tillers (Tanaka *et al*., 2015). Root branches are located underground, while tiller and inflorescence structures in rice undergo lateral branching above ground during the vegetative and reproductive stages, respectively. Tiller number is generally regarded as the determining factor of yield as tillers are specialized panicle‐bearing branches (Grillo *et al*., 2009). Several genes related to tiller development have been characterized; for example, *MOC1* is important in both tiller bud formation and outgrowth, while *OsTB1* negatively regulates axillary bud outgrowth (Li *et al*., 2003; Takeda *et al*., 2003). Moreover, both *MOC1* and *OsTB1* expression was found in the axillary meristem and tiller buds. *MOC1* was also detected at the leaf axils. The *LRK2* gene studied here was expressed in tiller buds and nodes, suggesting a role in tiller formation and outgrowth. In line with this, *LRK1* was previously found to increase both tiller and grain number (Zha *et al*., 2009). In the current study, overexpression of *LRK2* resulted in an increase in tiller number, but no significant change in the number of grains per panicle. Similarly, both *LRK1*‐ and *LRK2*‐overexpressing plants exhibited an increase in tiller number. These results suggest that *LRK2* and *LRK1* are expressed in different tissues, despite belonging to the same gene cluster. However, it is also possible that the diverse phenotypes of the transgenic lines were partly due to the different genetic backgrounds of the rice cultivars used for transformation (i.e. 9311 and Nipponbare).

Plants have evolved a number of morphophysiological and biochemical strategies at both the cellular and molecular levels to allow them to adapt to biotic and abiotic stresses. When plants encounter abiotic stresses, membrane‐localized receptors rapidly sense environmental signals and transmit them downstream, thereby activating stress‐related responses. During these processes, *LRKs* play important roles as both sensors and transducers (Lease *et al*., 1998; Shiu *et al*., 2004; Torii, 2004). Meanwhile, *RPK1* (receptor‐like protein kinase 1) is required for embryonic pattern formation and enhances both water and oxidative stress tolerance in *Arabidopsis* (Mandel *et al*., 2014; Masle *et al*., 2005; Meng *et al*., 2012; Shen *et al*., 2015; Shpak *et al*., 2004; Van Zanten *et al*., 2009). Similarly, *BAK1* (*BRI1*‐associated receptor kinase 1) was found to play a role in a number of diverse processes, including brassinosteroid signalling, the phytosulfokine (PSK) signal pathway, light responses, cell death and plant innate immunity (Chinchilla *et al*., 2007, 2009; Ingram, 2007; Ladwig *et al*., 2015; Li *et al*., 2002b; Nam and Li, 2002; Sun *et al*., 2013). In this study, the novel *LRK* gene *LRK2* was found to function in drought tolerance and tiller development. Identification and further elucidation of the molecular mechanisms of such genes would be valuable in rice production management and genetic improvement studies.

*EF1A* was first shown to function in protein synthesis, and since then has been implicated in a number of biochemical processes such as interactions with the cytoskeleton (Gross *et al*., 2005), apoptosis (Byun *et al*., 2009; Zhang *et al*., 2015) and cell proliferation (Sanders *et al*., 1992). *EF1A* also interacts with phospho‐Akt in breast cancer cells and regulates their proliferation, survival and motility (Pecorari *et al*., 2009), and is expressed ubiquitously in humans. Although a highly abundant cellular protein associated with the cytoskeleton, the function of *EF1A* in rice remains unknown. Phylogenetic analysis revealed a close genetic relationship between LRK2 and PSKR1 from *A. thaliana*. PSKR1 is a PSK receptor that stimulates plant growth and differentiation (Igarashi *et al*., 2012). In this study, the LRK2 intracellular domain was also found to directly interact with OsEF1A in double molecule fluorescence analysis and a yeast two‐hybrid assay. The findings further suggest that *LRK2* interacts with *OsEF1A* to regulate plant developmental processes such as cell proliferation, thereby increasing plant branching.

**Figure 8:**
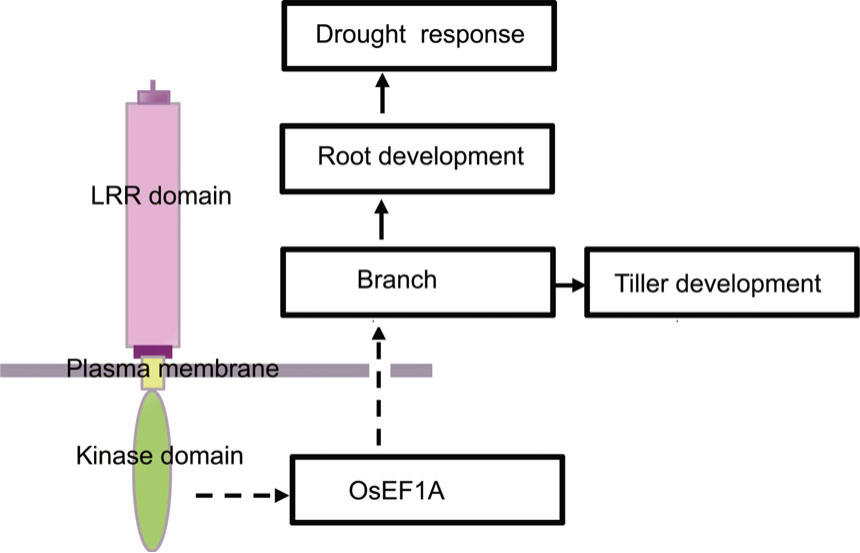
Proposed model of the role of the *LRK2* gene in drought tolerance and tiller development in rice. LRR: leucine‐rich repeat.

Based on the results of this study, a model was proposed to describe the functions of rice *LRK2* in regulating tiller size and the drought stress response (Figure 8). By interacting with other molecules such as the eukaryotic translation elongation factor, *LRK2* is thought to regulate cellular proliferation, promote branch development and subsequently increase tiller number. The larger root system subsequently contributes to an increase in drought tolerance. As rice is an important food crop and drought one of the main factors affecting crop growth and yield, methods aimed at increasing yield and creating drought‐resistant varieties are crucial. The findings of this study demonstrate the potential of *LRK2* as a useful tool for crop improvement, particularly with regard to drought tolerance, helping enhance agronomically useful traits such as tiller number, yield and the number of grains per plant. Future research will facilitate further improvements in abiotic stress tolerance in crops through genetic manipulation, thereby paving the way for a new green revolution.

## Experimental procedures

### Phylogenetic analysis

Phylogenetic analysis of LRK2 and other LRK proteins was carried out using MEGA 6.0 and a phylogenetic tree constructed using ClustalX 1.83 and UltraEdit21 software.

### Generation of transgenic rice

Full‐length cDNA of *LRK2* was amplified from the rice cultivar Nipponbare, and the confirmed sense and antisense sequences inserted into the *pCAMBIA 1300‐2 × 35S* vector under control of the cauliflower mosaic virus 35S promoter to produce *LRK2*‐overexpressing and knockdown lines. The primers 5′‐GTC GGTACCATGCAGCCACCTCATTCTTCATGCAAC‐3′ and 5′‐CAGGTCGAC TCAGTCGGAGCCTACACTGTCCAG‐3′ were used to construct *2 × 35S::LRK2*, and primers 5′‐GTCGGTACCTTATATCTTTATTTCAGTGCCTATACTGTC‐3′ and 5′‐CAGGTCGACATGCAGCTACTTCATTACAAGAAACACAG‐3′ to construct *2 × 35S::antiLRK2*. The constructs were transformed into Nipponbare mediated by Agrobacterium‐mediated transformation as described previously (Attia *et al*., 2005). The primers 5′‐CAAGACCTGCCTGAAACCGAACTG‐3′ and 5′‐GCGCGTCTGCTGCTCCATACA‐3′ were used to confirm the transgenic plants.

### Subcellular localization of rice *LRK2*

To investigate the subcellular localization of *LRK2*, the *LRK2* coding sequence was amplified with the cDNA clone as the template, using primers 5′‐CGGGGTACCATG CAG CCACCTCATTCTTCATGCA‐3′ and 5′‐TCCCCCGGGGTCGGA GCCTACACT GTCCAGGCAG‐3′. The PCR product was used to construct a *35S::LRK2‐eGFP* fusion plasmid, which was transformed into tobacco leaves by *Agrobacterium*‐mediated transformation, as described previously (Yang *et al*., 2000). The transformed plants were cultured at 22 °C under 16‐h light for 24–48 h. The GFP image was subsequently obtained using a Leica TCS SP5 AOBS confocal laser microscope.

### Promoter–GUS analysis

The *LRK2* promoter, an approximately 2000‐bp DNA fragment upstream of the translation start site, was amplified using primers 5′‐TTGAAGCTTCCTCCCACCTCCAAGTGTTCAAC‐3′ and 5′‐CCAGGATCCGGTTTTCTGGTGATACTAGCATGGAAG‐3′ from 9311. The DNA fragment was then cloned into the *pBI121* expression vector between the *Hin*dIII and *Bam*HI sites using the above transformation method. The primers 5′‐CTGGATCCGTAGATCTGAGGAACCGACGA‐3′ and 5′‐GAGGACGTCTCACACGTGGTGGTGGTGGT‐3′ were used to confirm the transgenic plants. A GUS assay was performed at various developmental stages as described previously (Jefferson *et al*., 1987).

### Total RNA isolation, RT‐PCR and real‐time quantitative PCR analysis (qRT‐PCR)

Total RNA extraction was performed using an RNeasy Plant Mini Kit following the manufacturer's instructions (Qiagen, Germany). Translation of RNA into cDNA was performed using a ReverTra Ace qPCR‐RT Kit following the manufacturer's instructions (TOYOBO, Japan). Amplification of genes from the cDNA template was performed using specific primers with high‐fidelity primeSTAR HS DNA Polymerase according the user's manual (TaKaRa, Japan). Diluted reaction products were used as templates for RT‐PCR and real‐time quantitative PCR analysis. The following *LRK2*‐specific primers were used for RT‐PCR: 5′‐GTCGGTACCATGCAGCCACCTCATTCTTCATGCAAC‐3′ and 5′‐CAGGTCGACTCAGTCGGAGCCTACACTGTCCAG‐3′, and the following specific primers for qRT‐PCR analysis: 5′‐TCAGCATCCAAAAAACAGTTGAAC‐3′ and 5′‐CTCTGGATCAGAGGTGAACGAAC‐3′. Each data point represents three replicates, and each experiment was repeated twice.

### Growth conditions

Rice seeds (*O. sativa* ssp. japonica cv. Nipponbare) and transgenic plants were sown in pots after 3 day of germination at 37 °C. All plants were grown under 28 °C/16‐h light and 25 °C/8‐h dark conditions at 75% relative humidity in a glasshouse. For gene expression analysis, the roots of Nipponbare rice seedlings at the three‐leaf stage were immersed in PEG6000 (20%) or water for 3, 6, 12 and 24 h. After treatment, the seedlings were harvested and used for total RNA isolation.

### Drought stress treatment

For drought stress treatment, 12‐day‐old *2 × 35S::LRK2*,* 2 × 35S::antiLRK2* and wild‐type plants were grown in pots under the indicated conditions then treated with 20% PEG6000, respectively, until the leaves of the wild type were rolled as a result of drought stress. Plants were then rewatered. The phenotypes of the plants were subsequently observed and photographed at various time points. After recovery, survival rates were calculated by counting plants with green healthy young leaves.

### Measurements of root activity

Root activity in terms of TTC reduction was measured as described previously (Hu *et al*., 2016; Steponkus and Lanphear, 1967). Fresh roots from wild‐type and transgenic plants were placed in six test tubes, each containing 2 mg sodium thiosulfate and 5 mL of various concentrations of TTC or distilled water, followed by the addition of 5 mL phosphate buffer (pH 7.5). The roots were incubated at 37 °C for 2 h after which 2 mL 1 m sulphuric acid was added to terminate the reaction. The roots were removed and ground in a mortar containing 3 mL ethyl acetate to extract the triphenylformazan (TTCH). TTCH was measured based on the absorbance of the supernatant at 485 nm. A standard curve was constructed with TTCH on the *x*‐axis and OD on the *y*‐axis. The root activity was determined by TTCH concentration for each fresh root as follows: root activity (mg g^−1^ h^−1^) = TTCH reduction (TTCH mg)/fresh root weight (FW g)/time (h). For each root activity measurement, data points represent the average of three replicates.

### Yeast two‐hybridization analysis

Yeast two‐hybrid library construction and screening was performed using BD Matchmaker library construction and screening kits (Clontech). The *LRK2D* coding region was fused in‐frame with the GAL4 DNA binding domain in the *pGBKT7* vector to generate the bait vector. Primers included *LRK2D‐F* (5′‐CTG CATATG CTTTTCTCGCTCAGGGATGC‐3′) and *LRK2D‐R* (5′‐CGC GTCGAC GTCGGAGCCTACACTGTCCAG‐3′). Rice ds‐cDNA, the vector *pGADT7‐Rec2* and bait construct *pGBKT7‐LRK2D* were then cotransformed into yeast strain AH109, which was subsequently plated directly onto SD/ Leu‐Trp‐His‐/50 mm 3‐AT medium followed by incubation at 30 °C for 4 days. Positive clones were screened according to the manufacturer's protocol. The *OsEF1A* coding region was cloned into pGADT7 and yeast two‐hybrid analysis performed using primers *OsEF1A‐F* (5′‐CCAGAATTCATGGGTAAGGAGAAGACGCACATCA‐3′), *OsEF1A‐R* (5′‐TTT GGATCCTTATTTCTTCTTGGCGGCAGCCTTG‐3′), *OsEF1A D1‐AD‐EcoRI‐F* (5′‐CCAGAATTCATTGTGGTCATTGGCCACG‐3′), *OsEF1A D1‐AD‐BamHI‐R* (5′‐TTTGGATCCGGGCTCGTTGATCTGGTCAAG‐3′), *OsEF1A D2‐AD‐EcoRI‐F* (5′‐CCAGAATTCGACAAGCCCCTACGTCTTCCC‐3′), *OsEF1A D2‐AD‐BamHI‐R* (5′‐TTTGGATCCGTCATCCTTGGAGTTGGAGGC‐3′), *OsEF1A D3‐AD‐EcoRI‐F* (5′‐CCAGAATTCGAGGCTGCCAGCTTCACCTC‐3′) and *OsEF1AD3‐AD‐BamHI‐R* (5′‐TTTGGATCCGATGACGCCAACAGCCACC‐3′). Yeast cells harbouring *pGBKT7‐LRK2D*+ *pGADT7‐OsEF1A*,* pGBKT7+ pGADT7‐OsEF1A*,* pGBKT7‐LRK2D+ pGADT7, pGBKT7+ pGBKT7, pGBKT7‐LRK2D+ OsEF1A‐ΔD3‐1*,* pGBKT7‐LRK2D+ OsEF1A‐ΔD3‐2*,* pGBKT7‐LRK2D+ OsEF1A‐D3‐1*,* pGBKT7‐LRK2D+ OsEF1A‐ΔD3‐3*,* pGBKT7‐LRK2D+ OsEF1A‐D3‐2* were selected on SD/Leu‐Trp‐ and SD/Leu‐Trp‐His‐/50 mm 3‐AT with X‐gal plates.

### Bimolecular fluorescence complementation assay

Bimolecular fluorescence complementation in tobacco was carried out as described previously (Schweiger and Schwenkert, 2014). *LRK2D* and *OsEF1A* cDNA fragments were amplified with the following primers *OsEF1A‐pSPYNE‐F* (5′‐CACACTAGTAT GGGTAAGGAGAAGACGCACAT‐3′), *OsEF1A‐pSPYNE‐R* (5′‐CGACCCGGGTT TCTTCTTGGCGGCAGCC‐3′), *LRK2D‐pSPYCE‐F* (5′‐CACTCTCGAATGCT TTTCTCGCTCAGGGATG‐3′) and *LRK2D‐pSPYCE‐R* (5′‐CGAGTCGACGTC GGAGCCTACACTGTCC‐3′). They were then cloned into the following split‐YFP vectors with nonoverlapping coding regions: *35S::SPYNE* and *35S::SPYCE* (Walter *et al*., 2004). The constructs were verified by sequencing and transformed, respectively, into *Agrobacterium*. *Agrobacterium* were grown at 28 °C to a final OD_600_ of 0.8 for agroinfiltration. Next, we mixed equal volumes of *Agrobacterium* culture carrying the constructs *OsEF1A‐pSPYNE* and *LRK2D‐pSPYCE*, selected the leaves of 3‐week‐old tobacco plants, and infiltrated the *Agrobacterium* suspension carefully into the tobacco leaves by pressing a syringe without a needle. Plants were watered and cultured at 22 °C under 16‐h light for 2 days. Images were collected using a Leica TCS SP5 AOBS confocal laser microscope.

## Conflict of interest

The authors declare no conflict of interests.

## Supporting information


**Figure S1** LRK2 promoter expression in transgenic rice.

**Figure S2** PCR analysis to confirm the transgenic lines.

**Figure S3** Gene expression levels in rice seedlings in response to drought treatment.

**Figure S4** Overexpression of *LRK2* increased the tiller number in rice.

**Table S1** Plant heights of control and transgenic lines.
