# Supplementary figures and images for: Overexpression of the leucine‐rich receptor‐like kinase gene LRK2 increases drought tolerance and tiller number in rice
Source: Plant Biotechnol J. 2017 Mar 23;15(9):1175–85. doi: 10.1111/pbi.12707 (PMC5552483; doi:10.1111/pbi.12707)

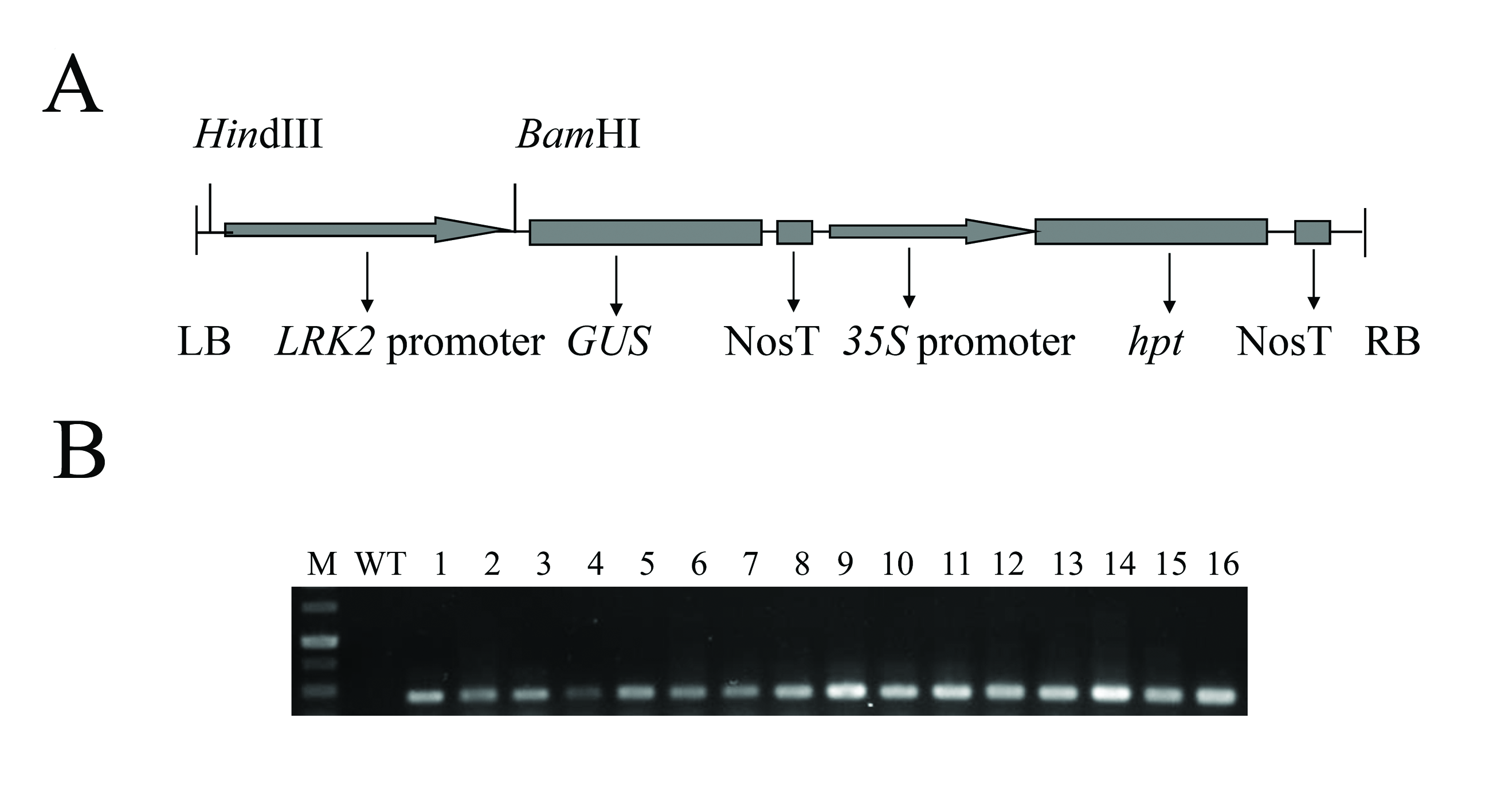

Supplement: Supplementary file 1 — Figure S1 LRK2 promoter expression in transgenic rice. [file PBI-15-1175-s004.tif]

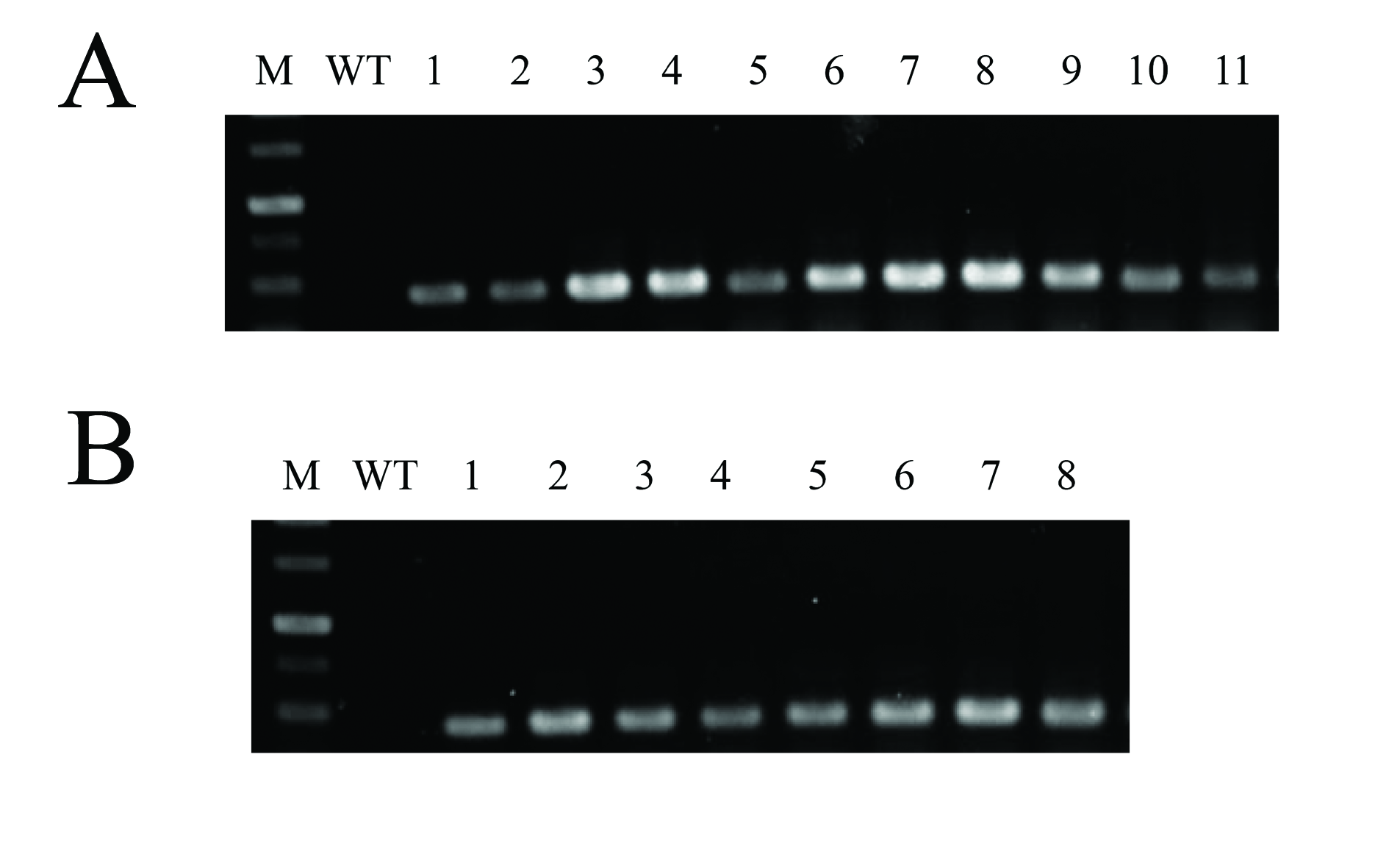

Supplement: Supplementary file 2 — Figure S2 PCR analysis to confirm the transgenic lines. [file PBI-15-1175-s003.tif]

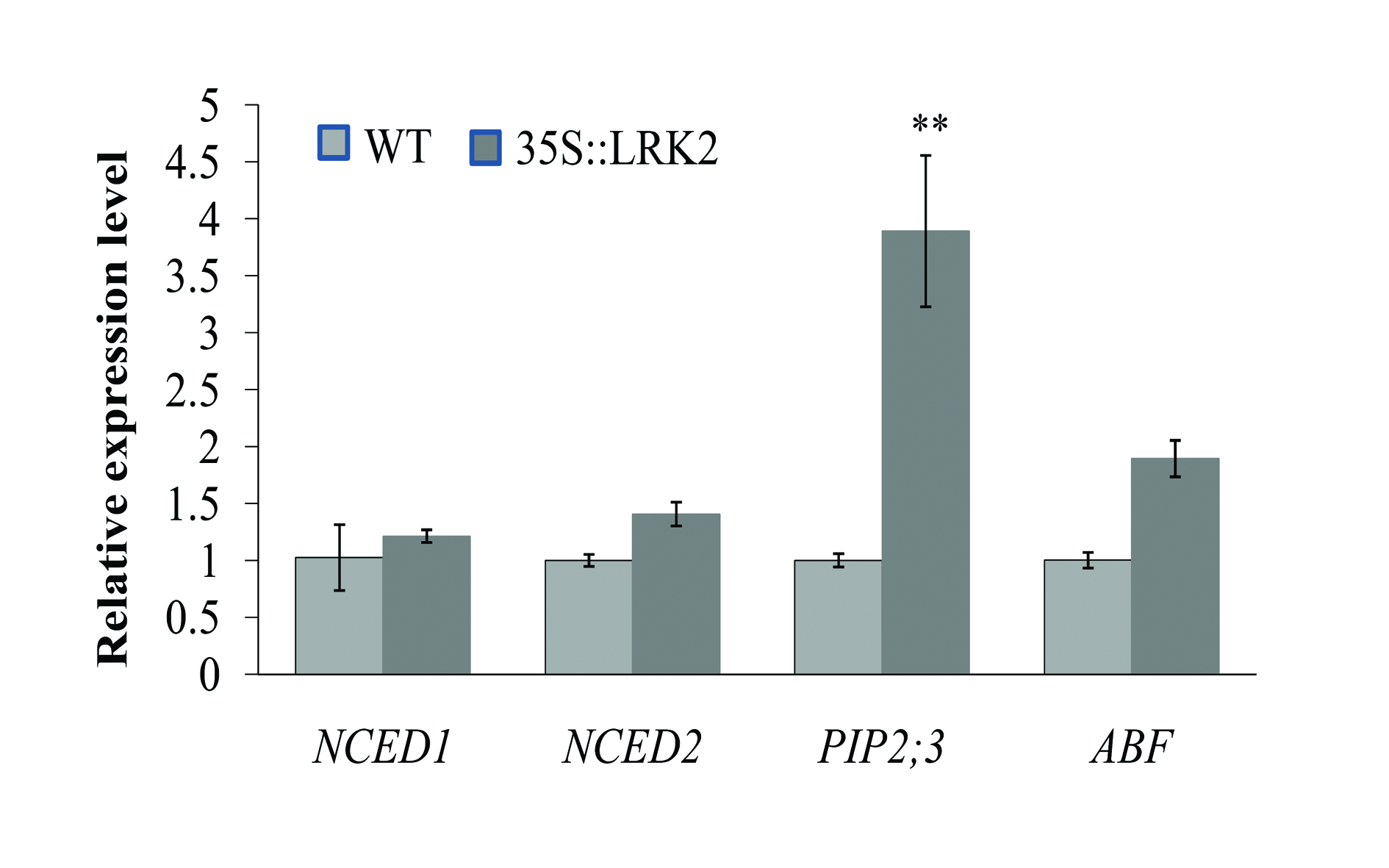

Supplement: Supplementary file 3 — Figure S3 Gene expression levels in rice seedlings in response to drought treatment. [file PBI-15-1175-s005.tif]

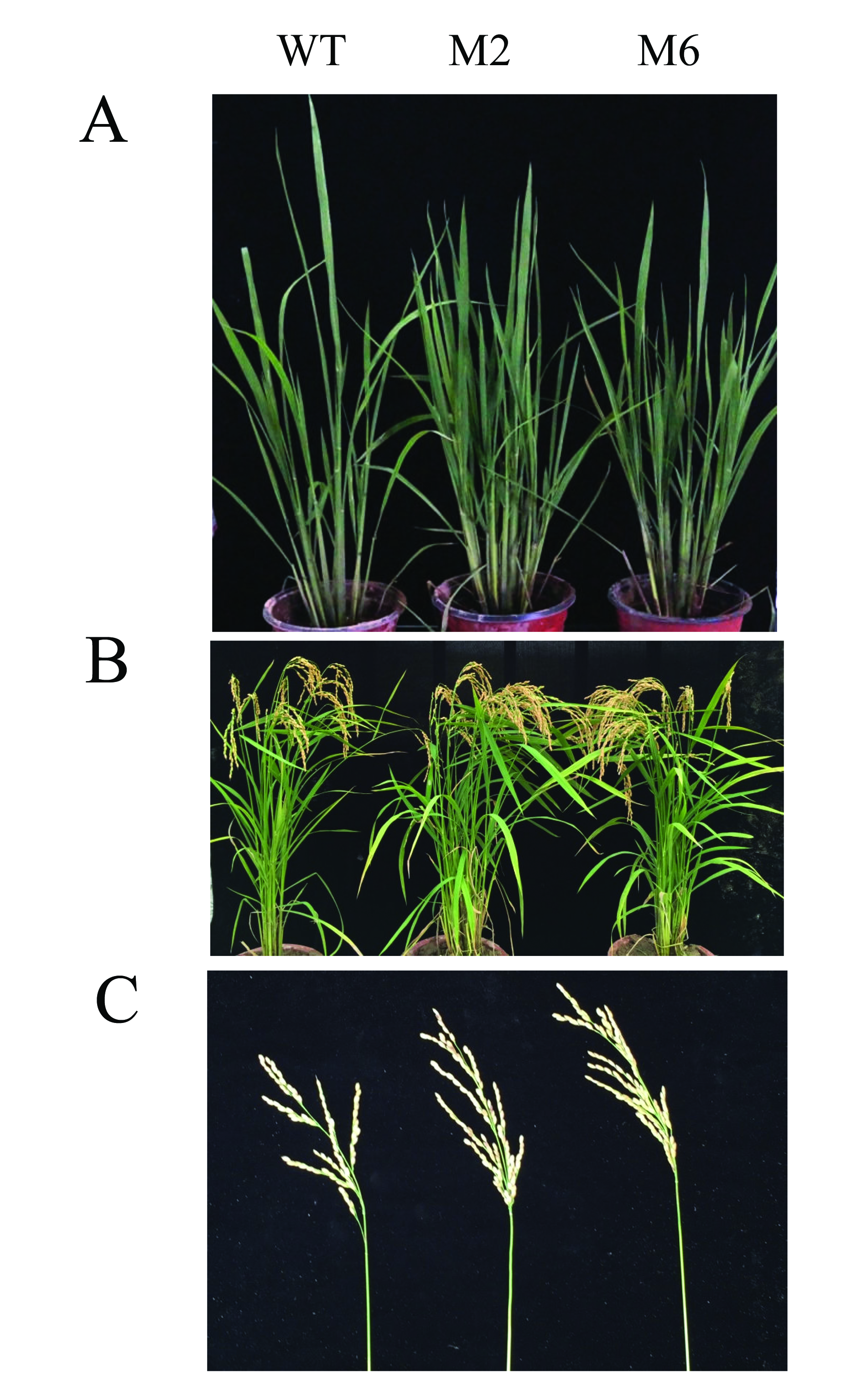

Supplement: Supplementary file 4 — Figure S4 Overexpression of LRK2 increased the tiller number in rice. [file PBI-15-1175-s001.tif]
