# Supplementary material for: Overexpression of the leucine‐rich receptor‐like kinase gene LRK2 increases drought tolerance and tiller number in rice
Source: Plant Biotechnol J. 2017 Mar 23;15(9):1175–85. doi: 10.1111/pbi.12707 (PMC5552483; doi:10.1111/pbi.12707)
Supplement: Supplementary file 5 — Table S1 Plant heights of control and transgenic lines. [file PBI-15-1175-s002.docx]

**Table S1.** Plant heights of control and transgenic lines.

| Plant type lines | Plant height |
| --- | --- |
| WT | 82.90 ± 4.93 |
| M2 | 74.55 ± 1.31* |
| M6 | 73.05 ± 3.58* |

Data were for random samples at plant maturity and are shown as mean ± SD for 20 individuals. **P* < 0.05, *t-test*.
